# Noisy quantum learning theory

**DOI:** 10.1038/s41467-026-73693-x

**Published:** 2026-05-29

**Authors:** Jordan Cotler, Weiyuan Gong, Ishaan Kannan

**Affiliations:** 1 Department of Physics, Harvard University, Cambridge, Massachusetts USA; 2 Harvard Quantum Initiative, Harvard University, Cambridge, Massachusetts USA; 3 John A. Paulson School of Engineering and Applied Sciences, Harvard University, Allston, Massachusetts USA

**Keywords:** Quantum information, Information theory and computation

## Abstract

While known quantum learning speedups operate in idealized noiseless regimes, coupling to uncharacterized systems is a noisy process even given fault-tolerant devices. Here we show that noise can eliminate exponential quantum advantages of unphysical, noiseless learners, while demonstrating more nuanced directions towards meaningful quantum speedups in noisy experiments. We introduce the complexity class ${\mathsf{NBQP}}$ ("noisy BQP”), modeling noisy fault-tolerant quantum computers that cannot generally error-correct the oracle systems they query. We prove that while natural ${\mathsf{NBQP}}$ learners may be exponentially weaker than their idealized counterparts, a superpolynomial gap remains between ${\mathsf{NISQ}}$ and fault-tolerant devices. Turning to canonical learning tasks, we find that the exponential advantage for purity testing collapses under local depolarizing noise. We then analyze noisy Pauli tomography, deriving lower bounds characterizing how instance size, quantum memory and noise jointly control sample complexity. We further study noise-dependent limitations on Heisenberg-limited metrology. Nevertheless, we identify a setting in which physical structure restores the purity testing speedup and highlight a noise-dependent polynomial speedup for Pauli tomography. Our results demonstrate that the primitives underlying quantum-enhanced experiments are fundamentally fragile to noise, and that realizing meaningful quantum advantages in future experiments will require interfacing noise-robust physics with available algorithmic techniques.

## Abstract (web summary)

Expected quantum advantages in learning unknown system properties usually rely on idealized scenarios. Here, the authors develop a computational complexity framework to rigorously describe situations in which a fault-tolerant quantum computer is available but its coupling to the system of interest is noisy, showing how retaining exponential advantage is nontrivial - but possible.

## Introduction

Quantum learning theory has advanced rapidly in recent years, and one of its key successes is recasting experimental protocols as learning problems, which admits a rich toolkit for determining when and how quantum computation-enhanced experiments can outperform conventional ones^1–13^. From this perspective, partially uncharacterized experimental systems serve as oracles providing quantum data, and the experimentalist implements a learning protocol that reveals the system’s properties through controlled interactions. When a quantum computer is available, it can be coherently coupled to the system of interest, enabling quantum computation on transduced data. In idealized settings, such quantum computation-enhanced experiments can provide a provable exponential advantage over conventional experimental protocols^9–11,14^.

These quantum speedups share two key characteristics. First, nearly all known idealized separations rely on learning tasks governed by intrinsically complex, high-weight structures, including states or dynamics that resist succinct classical representations^10,14–17^. For example, the quantum-enhanced sample-complexity gap in estimating physically motivated Pauli observables grows with the weight of the observables^16^, and classical simulation of quantum dynamics becomes intractable when high-weight Paulis dominate the evolution^18,19^.

Second, exponential quantum advantages achieved through multi-copy measurements typically require entanglement across a number of qubits that scales extensively with the problem size^10,14,20^. Operationally, such algorithms are built from a small set of standard primitives, most notably maximally entangled (Bell-basis) measurements and many-qubit SWAP operations, that underlie idealized superpolynomial speedups in quantum learning, property testing, and computation^21–24^.

For most known examples of quantum experimental advantage, the quantum computer and its coupling to the experimental system are assumed to be noiseless. While a noisy quantum computer can be error-corrected, the experimental system given to us by Nature does not come embedded in an error-correcting code. We are therefore in a setting where an error-corrected quantum computer can only access the experimental system via a noisy coupling – one that must also mediate between the logical encoding of the quantum computer and the bare physical degrees of freedom of the experimental system. Moreover, since the experimental system corresponds to a partially unknown state or channel, it cannot generally be embedded into an error-correcting code by the experimentalist without additional overhead^25,26^. Noise thus fundamentally changes the character of quantum computation-enhanced experiments and may obviate many of their known advantages.

In this paper, we develop a theory of noisy quantum learning: for several prominent examples of quantum computation-enhanced experiments, we establish when noise eliminates quantum advantages and when such advantages persist.

Concretely, noisy quantum learning theory reveals two distinct mechanisms by which noise erodes ideal quantum advantages, tied to the same characteristics that enable them. Namely, our results show that the same high-complexity components that enable exponential entanglement-enabled separations to form in the noiseless setting are the most vulnerable to noise. As a result, the very structures that underwrite ideal quantum advantages also provide the primary mechanism through which noise destroys them. Moreover, in our noisy setting, implementing the canonical SWAP and Bell-measurement primitives on noisy, uncharacterized quantum systems leads to an exponential blow-up in sample complexity that cannot be circumvented by any adaptive learning strategy. These results pertain to a broad framework which we introduce to model noisy quantum experiments, in which the learner is subjected to repeated depolarizing noise upon querying natural oracle systems but is permitted to use any (possibly adaptive) strategy that can include quantum error-correction. Absent additional structure in the physical system that can be exploited for error correction, the primitives that enable ideal quantum speedups cease to provide robust advantages in the noisy regime.

Given that several canonical results in quantum learning theory break down in noisy settings, what kinds of quantum advantages can still persist in the presence of such noise? Our work suggests two avenues to realize practically meaningful gains in noisy quantum-enhanced experiments. Many physical systems possess endogenous robustness: thermalizing systems have built-in noise resilience^27–29^, and long-range correlations in many-body quantum systems can be protected by renormalization group structure^30–32^. We provide illustrative examples of when the robustness of the physical system and the fault-tolerant architecture of the quantum computer can meet in the middle, allowing exponential quantum advantages to survive. Even when asymptotic exponential separations are impossible, we show that noise-dependent polynomial advantages can survive. We demonstrate that by tracking how resource costs for tomography and metrology are jointly controlled by noise rates, quantum resources, and instance size, we can rigorously demonstrate large speedups for noisy learning tasks at finite sizes.

## Results

**Fig. 1:**
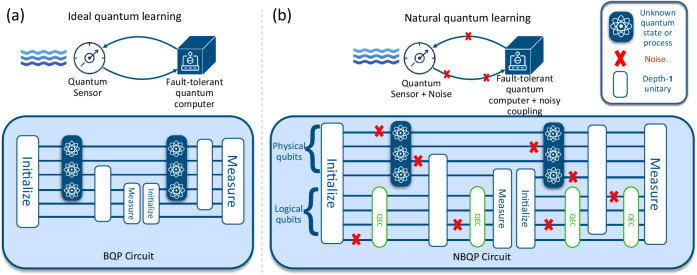
Schematic of idealized and natural quantum experiments. **a** *Ideal quantum learning*: a noiseless quantum computer queries an unknown quantum state or channel through a sensor, yielding noiseless coherent oracle access modeled by BQP^*O*^. **b** *Natural quantum learning*: a noisy but fault-tolerant quantum computer queries an unknown quantum state or channel through a sensor, with noisy coupling occurring at the physical-qubit level. The quantum circuit must correct errors on the quantum computer and contend with noise induced by coupling to the experimental system, modeled by ${{\mathsf{NBQP}}}^{O}$.

We first introduce the complexity class NBQP corresponding to noisy but fault-tolerant quantum computers with a constant error rate per qubit per circuit layer. The noise rate is taken to be below the threshold for fault-tolerant error correction, so that NBQP = BQP. Accordingly, we refer to learning protocols captured by BQP^*O*^ as ideal quantum learning, and those captured by NBQP^*O*^ as *natural quantum learning*, which are depicted in Fig. 1. This is crucially distinct from^12^, in which noisy quantum computers incapable of fault-tolerant error correction were formalized as the complexity class NISQ, and it was shown that there exist oracles *O* for which NISQ^*O*^ ⊊ BQP^*O*^, meaning that even adaptive NISQ protocols are superpolynomially slower than noiseless quantum computers for certain learning problems. Using oracle constructions from^12^ and^33^, we prove two superpolynomial oracle separations: ${{{\rm{NBQP}}}}^{{O}_{1}}\subsetneq {{{\rm{BQP}}}}^{{O}_{1}}$ and ${{{\rm{NISQ}}}}^{{O}_{2}}\subsetneq {{{\rm{NBQP}}}}^{{O}_{2}}$.

Next, we move from complexity separations to concrete noisy learning tasks. We begin by studying the NBQP complexity of testing the purity of a quantum state, showing that an ideal exponential quantum advantage is fundamentally obstructed by noise unless the class of states in question has a latent error-correcting structure. We give a concrete example of such a structure in a physically-motivated toy setting: assessing the purity of a black hole microstate in the bulk of a tensor-network model of holographic duality using only noisy measurements of the boundary state. Next, we analyze Pauli shadow tomography, demonstrating a tradeoff between sample complexity, the noise rate, and the complexity of the estimated observables. Using similar techniques, we show that error-correction-based metrology protocols retain Heisenberg-limited sensitivity only up to a sensing timescale that scales inverse-polynomially with sensor dimension and noise rate. Proving these lower bounds requires ruling out any adaptive strategy that mitigates or circumvents the noise. For this, we leverage the learning tree formalism introduced in^10^. While it is still possible to have a meaningful advantage of certain quantum-enhanced experiments vis-à-vis conventional experiments in the noisy setting, we find that noise exponentially degrades the most prominent idealized quantum advantages.

### Oracle separations between ${\mathsf{NBQP}},\,{\mathsf{BQP}}$, and ${\mathsf{NISQ}}$

We now make precise the notions introduced above. An ideal quantum computer operates as if each qubit evolves noiselessly between operations; in practice, errors accumulate at each circuit layer. Fault-tolerant quantum computation tells us these errors can be corrected if the error rate per qubit per circuit layer is below a certain threshold, motivating the following complexity class.

#### Definition 2.1

(NBQP complexity class, informal) ${\mathsf{NBQP}}$ contains all problems solvable in polynomial time by a noisy quantum computer with polynomial-size circuits, where all operations are subject to constant depolarizing noise per qubit at a rate below the threshold of known quantum fault-tolerance schemes.

In particular, the ${{\mathsf{NBQP}}}$ model assumes that after every round of parallel logical operation with two-qubit gates, each qubit is hit with independent local noise of constant strength. A problem is in NBQP if it is solvable within this circuit model for any constant noise rate above 0. Unlike previous work characterizing the computational power of NISQ devices^12^, our model allows a polynomial-size register of ancilla qubits that can be used to perform mid-circuit measurements. We work with depolarizing noise for concreteness, as it is a standard and well-studied error model. Our lower bounds hold for any noise model at least as strong as depolarizing noise, and our constructions can be adapted to other local stochastic noise models with constant error rate per qubit, such as adversarial noise. In physical applications where hardware leads to weaker noise models, such as local dephasing, it is possible that additional problem structure that exploits noise-robust quadratures can maintain meaningful computational speedups.

Under the above definition, an ${\mathsf{NBQP}}$ quantum computer can use mid-circuit state preparation and measurement to implement error correction after each layer of gates on any register embedded into a code. The threshold theorem^34^ then implies ${\mathsf{NBQP}}={\mathsf{BQP}}$. However, the situation changes when we introduce Oracle access. To make this concrete, consider coupling a fault-tolerant quantum computer to a partially uncharacterized quantum material in the laboratory. Even if the quantum computer itself is fully error-corrected, the interaction with the material occurs at the physical-qubit level and is therefore noisy. We cannot simply transduce the material’s state into an error-correcting code; even if this were technologically feasible, the encoding procedure would partially corrupt the very quantum information we are trying to learn about. The resulting asymmetry — that errors on the computation can be corrected, but errors on oracle queries cannot — opens the possibility of nontrivial separations between ${{\mathsf{NBQP}}}^{O}$ and ${{\mathsf{BQP}}}^{O}$. While error mitigation may be possible for particularly structured oracles, we demonstrate that the inability to perform true error correction on the unknown system can starkly degrade our ability to learn from experiments, leading to superpolynomial oracle separations between ${\mathsf{NBQP}}$ and ${\mathsf{BQP}}$.

#### Theorem 2.2

There exists an oracle *O*_1_ such that ${{\mathsf{NBQP}}}^{{O}_{1}}\subsetneq {{\mathsf{BQP}}}^{{O}_{1}}$.

To prove this separation, we adapt the lifted Simon oracle construction of^12^. Conceptually, the standard Simon oracle is modified so that slight perturbations to its input result in completely uninformative outputs, making the oracle highly nonrobust to NBQP-type noise. More concretely, given a function *f*: {0, 1}^*n*^ → {0, 1}^*n*^, consider the usual Simon’s promise: either (i) *f* is injective, or (ii) there exists a nonzero *s* ∈ {0, 1}^*n*^ such that *f*(*x*) = *f*(*x* ⊕ *s*) for all *x* (a Simon’s function). The lift $\widetilde{f}$ acts on 2*n* input bits but is nontrivial only when the last *n* bits are 0^*n*^. A noiseless quantum computer can simply restrict its queries to strings of the form *x* 0^*n*^ and then run the standard Simon’s algorithm^35^ on the first *n* bits. Thus, the promise problem, which asks whether *f* is injective or a Simon’s function, lies in ${{{\rm{BQP}}}}^{{O}_{1}}$ with *O*(*n*) queries.

For ${{{\rm{NBQP}}}}^{{O}_{1}}$, oracle calls act on physical qubits, and in our model, each such call is preceded and followed by a layer of depolarizing noise. Even if the algorithm tries to prepare “good” queries of the form *x* 0^*n*^, these noise layers quickly flip some of the trailing *n* bits, so with high probability the actual query lies outside the special subspace on which $\widetilde{f}$ encodes *f*; on those inputs, *O*_1_ simply outputs 0. In effect, to a noisy learner, *O*_1_ is almost indistinguishable from a trivial oracle, and exponentially many queries in *n* are required for such a learner to tell the difference. Using the hybrid/distinguishability framework of^12^, namely their channel-level hybrid lemma and a node-perturbation argument in our learning-tree model (formalized as Supplementary Lemmas D.16 and D.17), we show that any *λ*-noisy circuit making *N* oracle calls has output distribution within *N*^2^*e*^−Ω(*λn*)^ total variation distance of the distribution obtained by replacing every oracle call with the identity channel. Under this identity oracle, the two promise cases (injective versus Simon’s *f*) induce exactly the same distribution, so any ${{{\rm{NBQP}}}}^{{O}_{1}}$ algorithm with *N* = poly(*n*) cannot achieve constant distinguishing advantage, whereas a ${{{\rm{BQP}}}}^{{O}_{1}}$ algorithm can. This yields the strict separation ${{{\rm{NBQP}}}}^{{O}_{1}}\subsetneq {{{\rm{BQP}}}}^{{O}_{1}}$ claimed in the theorem.

Despite this limitation, an NBQP machine can implement polynomial-depth fault-tolerant quantum circuits, which are believed to be strictly more powerful than the logarithmic-depth circuits achievable by noisy intermediate-scale (NISQ) devices^36–39^. Leveraging this gap in coherent depth, we prove a superpolynomial oracle separation between NISQ and ${\mathsf{NBQP}}$, showing that fault-tolerant learners retain an advantage over near-term devices even when all access to the experimental system is noisy.

#### Theorem 2.3

There exists an oracle *O*_2_ such that ${{\mathsf{NISQ}}}^{{O}_{2}}\subsetneq {{\mathsf{NBQP}}}^{{O}_{2}}$.

For the proof of this theorem, we explicitly leverage the fundamental gap between NISQ and NBQP machines: the latter has the ability to perform quantum error correction, and thus can execute deep quantum circuits. Following^33^, we start from the *d*-level Shuffling Simon’s Problem, a variant of Simon’s problem challenging for shallow quantum circuits to solve. Here, a Simon function *f* on *n* bits is embedded and randomly permuted inside a much larger domain so that only a hidden subset of inputs carries any information about the secret *s*. We then define an encoded shuffling oracle ${{{\mathcal{O}}}}_{f,d}^{{{\rm{enc}}}}$ that acts like this shuffled Simon oracle on a logical code space of a fixed fault-tolerant quantum error correction scheme, and trivially outside it. By Theorem 4.11 of^33^, there is a noiseless depth-*O*(*d*) circuit that recovers *s*, and an NBQP machine can simulate this circuit fault-tolerantly (for the noise rate *λ* below threshold) with only polynomial overhead, and so ${\mathsf{Enc}}-d-{\mathsf{SSP}}\in {{{\rm{NBQP}}}}^{{O}_{2}}$.

The lower bound against ${\mathsf{NISQ}}$ proceeds in two steps. First, building on the analysis of^33^, we show that any ${{\mathsf{BPP}}}_{d}^{{\mathsf{QNC}}}$ algorithm has exponentially small success probability on the encoded problem: even if it makes polynomially many depth-*d* quantum queries, the domain in which the Simon’s function is embedded is so large that the algorithm is very unlikely to query the relevant subspace. While classical advice between bounded-depth subroutines can reveal successively more information about this “hidden domain", the number of permutations applied to Simon’s function is, by construction, too large for any ${{\mathsf{BPP}}}_{d}^{{\mathsf{QNC}}}$ algorithm to locate the domain. Mathematically, we carry out this argument by demonstrating that, with high probability, all oracle queries made by the algorithm can be replaced by “shadow” queries that agree with the true oracle outside a small wrapper set containing the hidden domain, but output no information on the domain itself; even after this swap, the output distribution of the algorithm is hardly altered. Upon this replacement, the algorithm learns nothing about *s*, as it no longer has access to the embedded Simon’s function (Supplementary Lemma D.11), and can thus do no better than random guessing. This establishes an exponentially small success probability for any ${{\mathsf{BPP}}}_{d}^{{\mathsf{QNC}}}$ algorithm.

Second, we show that any ${\mathsf{NISQ}}$ algorithm making polynomially many oracle calls can be simulated, up to vanishing total variation distance, by such a ${{\mathsf{BPP}}}_{d}^{{\mathsf{QNC}}}$ algorithm: we cut each noisy circuit at a fixed depth threshold and use KL-divergence bounds from^12^ to argue that replacing deeper noisy circuits by shallow ones only changes the leaf distribution of the learning tree by *o*(1). Combining these two ingredients and applying Le Cam’s two-point method, we obtain that no ${\mathsf{NISQ}}$ algorithm with poly(*n*) queries can recover Simon’s secret with success probability at least 2/3, whereas an ${\mathsf{NBQP}}$ algorithm can, yielding the oracle separation ${{\mathsf{NISQ}}}^{{O}_{2}}\subsetneq {{\mathsf{NBQP}}}^{{O}_{2}}$.

### Purity testing in noisy quantum experiments

So far, our results have established oracle separations between relativized complexity classes. We now turn to more concrete noisy learning tasks, asking how sample complexity scales with coherent quantum memory and access to joint measurements, and whether exponential quantum advantages can survive constant local noise. Our first result in this direction, Theorem 2.4 addresses a canonical task in quantum learning theory and property testing, namely testing the purity of a quantum state, which has been highlighted as one of the few examples exhibiting quantum advantages in both sample and computational complexity^9–11,14,16,20,24,40^. In the ideal setting, distinguishing an *n*-qubit maximally mixed state from a fixed pure state requires Ω(2^*n*^) samples, whereas permitting joint measurements on two copies reduces the task to only *O*(1) samples and constant computational time. This separation is powered by a quantum subroutine that appears throughout the literature on quantum speedups in learning, property testing, and computation: the multi-qubit ${\mathsf{SWAP}}$ operation^14,21–24,41^. Here, we show that this super-exponential quantum speedup is completely degraded by only a single layer of noise.

#### Theorem 2.4

(No quantum advantage for purity testing in the presence of noise, informal) Any algorithm that can test the purity of a quantum state using noisy two-copy measurements requires at least order *c*(*λ*)^*n*^ samples, where *c*(*λ*) > 1 is a constant depending only on the noise rate.

Our proof begins with the following hypothesis testing problem: given copies of a state *ρ*, guaranteed to be either a maximally mixed state or a fixed pure state sampled from the Haar measure on *n*-qubits, can we identify the ground truth with probability at least 2/3? We then construct a learning tree model for algorithms which can make adaptive, joint measurements on *ρ* ⊗ *ρ*, interleaved with classical computation. As in the NBQP model, each pair *ρ* ⊗ *ρ* is corrupted by a depolarizing channel ${{{\mathcal{D}}}}_{\lambda }^{\otimes 2n}$ before performing an *arbitrary* 2*n*-qubit POVM; note that the latter part permits noiseless quantum circuits of any depth. Hence, our lower bound holds against any model of noisy computation stronger than a single layer of depolarizing noise applied at the outset.

Our analysis proceeds by bounding the likelihood ratio between the leaf distributions generated by this learning tree when its measurement outcomes arise from the maximally mixed state versus from a fixed, Haar-random pure state. To do so, we focus on a fixed root-to-leaf path, utilizing the tree’s multilinear structure to reinterpret the learning problem. Under our reinterpretation, it suffices to bound the concentration of the output distributions of intermediate nodes in the tree, which we achieve by proving several technical lemmas regarding the noise channel and using the martingale formalism from^16^. Because each node concentrates by a factor exponentially small in the noise rate, every experiment is highly uninformative, and we obtain our lower bound.

While we have shown that assessing the purity of a generic quantum state is fundamentally obstructed by noise, there are natural classes of noise-robust states for which purity testing remains feasible. A toy but instructive example arises in tensor-network models of the AdS/CFT correspondence^42^, such as the HaPPY code^30^. In essence, a HaPPY tensor network *T* defines an isometric encoding $T:{{{\mathcal{H}}}}_{{{\rm{bulk}}}}\to {{{\mathcal{H}}}}_{{{\rm{bdy}}}}$ from a collection of bulk logical qudits on a finite hyperbolic lattice to boundary physical qudits arranged on a one-dimensional ring. In the AdS/CFT interpretation, ${{{\mathcal{H}}}}_{{{\rm{bulk}}}}$ models a code subspace of low-energy quantum-gravitational degrees of freedom on a spatial slice of AdS, while ${{{\mathcal{H}}}}_{{{\rm{bdy}}}}$ models the Hilbert space of the dual CFT on the boundary circle. The quantum error-correcting structure of *T* discretely implements bulk-boundary reconstruction: local bulk operators can be reconstructed on many overlapping boundary regions, and the radial direction of the network plays the role of a renormalization group scale^43^.

**Fig. 2:**
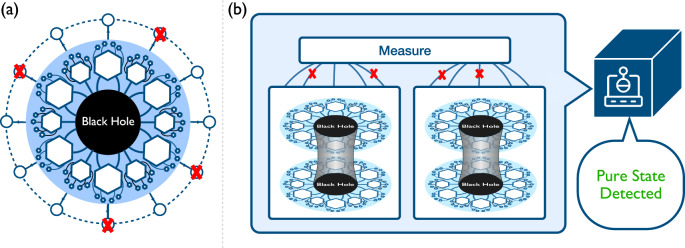
Example of physical structure recovering learning advantage. **a** *Holographic encoding of a black hole*: a HaPPY tensor network with a central bulk region encoding a black hole state, shown as a black disk. The outer dangling legs represent boundary qubits of the dual CFT; red crosses indicate qubits lost to an erasure channel acting independently on each boundary site. **b** *Quantum-enhanced purity test*: two noisy boundary copies are first approximately decoded back toward the bulk and then used as input to a joint measurement (e.g. a two-copy SWAP test) implemented by a quantum device. This protocol distinguishes whether the bulk black hole state is pure or mixed using only a constant number of copies, even in the presence of boundary erasures.

In our setting, we contract all bulk legs with $| 0\rangle$ states except those on an inner disk, and we use the remaining bulk legs on that disk to model a black hole (see Fig. 2a). Concretely, on this inner disk we insert a state *ρ*_BH_ on the corresponding bulk Hilbert space, which is either a fixed Haar-random pure state (a single microstate) or the maximally mixed state (a toy microcanonical ensemble). The resulting boundary state is then a holographic encoding of either a pure or mixed black hole in the bulk, and our operational task is to decide, from noisy measurements of the boundary CFT degrees of freedom alone, which case holds. Because the black hole degrees of freedom are protected by the HaPPY code and only indirectly exposed to noise through the boundary, a suitably designed SWAP-test-type procedure acting on (approximately) decoded bulk modes (see Fig. 2b) is partially robust to noise, and we show that this protection suffices to recover a quantum advantage. This is captured in the theorem below.

#### Theorem 2.5

(Purity testing for holographic black holes in the HaPPY code, informal) Consider a holographic HaPPY tensor network of total radius *R* with no uncontracted bulk legs, and remove all tiles within a smaller radius *r*, so that the resulting uncontracted bulk legs are replaced by either a fixed Haar-random pure state (a toy black hole microstate) or the maximally mixed state on the corresponding bulk Hilbert space (a toy black hole microcanonical ensemble). Given copies of the resulting boundary state, there exists a quantum-enhanced protocol which, even in the presence of constant-strength erasure error on every qubit at each circuit layer, uses only a constant number of copies together with joint measurements to distinguish these two cases with high probability. By contrast, any conventional experiment restricted to single-copy measurements requires at least ${2}^{\exp (\Omega (r))}$ copies to do so.

This result should be viewed as a holographic counterpart of our noisy purity-testing lower bound. On the one hand, the HaPPY code converts local boundary erasures into highly suppressed logical errors on the bulk black hole degrees of freedom: as long as $R\gtrsim r+O(\log r)$ and the erasure rate is below threshold, a greedy decoder can approximately recover *ρ*_BH_ from the noisy boundary state with failure probability that is exponentially small in 4^*R*−*r*^. More broadly, it is believed (but not known) that HaPPY-type holographic codes may admit fully fault-tolerant realizations against local noise^43–45^; in our setting, we only appeal to their rigorously understood erasure-correction properties. Composing the decoding map with a two-copy SWAP test on the recovered bulk region therefore reproduces, up to small decoding errors, the ideal two-copy purity test and yields a constant-copy quantum protocol. On the other hand, any protocol restricted to single-copy measurements on the boundary reduces, via bulk-boundary isometry and data-processing, to single-copy purity testing on an *L*_*r*_-qubit system, where *L*_*r*_ = Θ(4^*r*−1^) is the number of bulk legs in the excised region. Our general lower bound for noisy single-copy purity testing then implies a sample complexity of order ${2}^{\Theta ({L}_{r})}={2}^{\exp (\Theta (r))}$, establishing the separation claimed in the theorem.

Our HaPPY code example illustrates the first of two strategies for useful noisy quantum learning, namely targeting physical systems with latent error-resilient properties.

### Noise-dependent advantage in Pauli shadow tomography and metrology

**Fig. 3:**
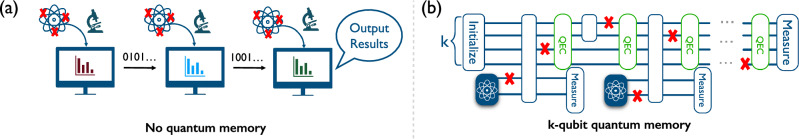
Noisy quantum learning with memory. **a** *Memoryless protocol*: each noisy copy of the unknown state is measured immediately, and only classical bit strings are stored, so different copies are never jointly entangled in the device. **b** *Protocol with k-qubit quantum memory*: a register of *k* qubits is initialized, repeatedly interacts with fresh noisy copies of the state, and is stabilized by intermittent QEC cycles (green boxes) before a final joint measurement. Red crosses indicate local noise events on the physical qubits.

To further understand how noise reshapes quantum learning advantages, we now consider the well-studied problem of Pauli shadow tomography, namely, estimating expectation values of (potentially mutually noncommuting) Pauli observables, which in ideal settings exhibits an exponential memory-sample tradeoff^10^. Any noiseless conventional protocol restricted to single-copy measurements, or to *k* < *n* qubits of quantum memory, requires a number of samples exponential in *n* − *k*, whereas a quantum-enhanced learner with two-copy access (i.e. *n* ancillary qubits of quantum memory) succeeds with only *O*(*n*) samples using Bell-basis measurements; these two settings are depicted in Fig. 3a (memoryless or small-memory single-copy experiments) and Fig. 3b (architectures with a quantum memory register enabling multi-copy measurements). In Theorem 2.6, we refine this separation in the NBQP setting, quantifying the noise-dependence of lower bounds with and without ancillary quantum memory. Notably, even when *n* qubits of memory are provided, enabling two-copy measurements, we show that order (1−*λ*)^−*n*^ samples are necessary. We give a noisy 2-copy algorithm achieving this scaling up to a constant factor in the exponent, establishing a quantum advantage in noisy Pauli tomography that depends polynomially on the noise rate, and negating the exponential speedup of ideal Bell-measurement-based strategies.

#### Theorem 2.6

(Complexity of noisy Pauli shadow tomography, informal) In the presence of constant depolarizing noise per qubit, any quantum algorithm without ancillary quantum memory which can identify a Pauli-structured state with high probability requires order 2^*n*^*f*(*λ*)^*n*^ measurements, where *f*(*λ*) ∈ [1, *∞*) for *λ* ∈ [0, 1]. Given an additional *k *≤ *n* qubits of quantum memory, Ω(2^*n*−*k*^(1−*λ*)^−*n*^) samples are still required. When *k* = *n*, there exists a quantum-enhanced learning algorithm with access to noisy two-copy measurements solving the Pauli-identification task using $\widetilde{O}({(1-\lambda )}^{-4n})$ samples.

As an immediate corollary, we find a sample complexity separation between our two-copy algorithm and any single-copy strategy for the same Pauli identification task. This separation depends on the noise rate, and interpolates smoothly between the ideal exponential separation for Pauli tomography and a polynomial (in the noise rate) separation for Θ(1) local noise. A related separation is presented in Ref. ^46^; that work considers diamond-norm gate noise and access to perfect state copies, a weaker noise model than NBQP, and gives a lower bound which considers only noiseless conventional experiments.

#### Corollary 2.7

(Noise-dependent quantum advantage in Pauli identification) Let *N*_*SC*_ be the optimal sample complexity for any single-copy algorithm for the *n*-qubit Pauli identification task from Theorem 2.6, and let *N*_*TC*_ be the sample complexity of the two-copy algorithm given in Theorem 2.6. Then ${N}_{SC}/{N}_{TC}=\Omega \left(a{(\lambda )}^{n}\right)$, where *a*(*λ*) is a function of noise rate such that up to a critical threshold λ^*^ ≈ 0.2, the provable advantage obeys the following scaling regimes:

1. $${N}_{SC}/{N}_{TC}=\exp (\Omega (({\lambda }^{*}-\lambda )n))$$ for *λ* = λ^*^ − Θ(1)

2. *N*_*SC*_/*N*_*TC*_ = Ω(poly(*n*)) for $\lambda={\lambda }^{*}-\Theta (\log n/n)$

3. *N*_*SC*_/*N*_*TC*_ = Ω(1) for *λ* = λ^*^ − *O*(1/*n*)

**Fig. 4:**
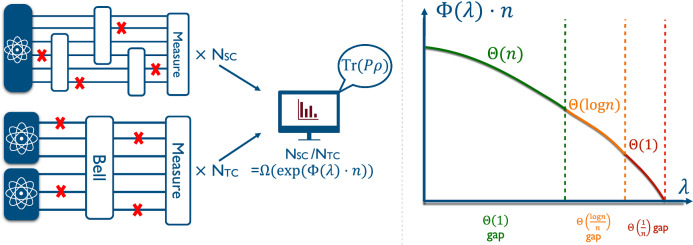
Noise-dependent quantum advantage in Pauli shadow tomography. The noise-aware quantum advantage of Corollary 2.7 is depicted. Letting *N*_*SC*_ denote the sample complexity of any single-copy noisy strategy for Pauli shadow tomography and *N*_*TC*_ denote the sample complexity of the noisy Bell sampling algorithm, the quantum advantage given by the ratio *N*_*SC*_/*N*_*TC*_ scales as $\exp (\Phi (\lambda )\cdot n)$ for a function Φ of the noise rate, where the exponent is plotted for fixed *n* and variable *λ*. Below a constant noise threshold λ^*^ ≈ 0.2, the advantage scales exponentially in *n* when *λ* is a constant gap smaller than λ^*^, polynomially in *n* for $\lambda={\lambda }^{*}-\Theta (\log n/n)$, and constant for *λ* = λ^*^ − *O*(1/*n*). Beyond λ^*^, the provable advantage is not meaningful, but this lies beyond practical fault-tolerance thresholds.

This is depicted pictorially in Fig. 4.

Our lower bounds span three models for quantum learning algorithms utilizing single-copy measurements. Namely, we consider (i) a learner without any ancillary quantum memory, (ii) one with *k* < *n* qubits of quantum memory, but where the memory must be reset after a constant number of queries and classical advice may be passed between experiments, and (iii) *k*≤*n* qubits of memory with unbounded lifetime. To utilize the learning tree formalism, we introduce another hypothesis testing problem (the Pauli identification task discussed above): given copies of the state $\rho=({\mathbb{1}}+P)/{\mathsf{tr}}({\mathbb{1}}+P)$, can we distinguish between the case where *P* is sampled uniformly from all 4^*n*^ − 1 non-identity Paulis and where *P* is the *n*-qubit identity matrix?

We next define learning trees for each experimental model. For models (i) and (ii), the output of each node of the tree is a classical bitstring. For both, we show that the distribution over bitstrings induced by the results of each experiment, given access to a ${\mathbb{1}}+P$-type state, concentrates around the distribution resulting from experiments which instead query a maximally mixed state. Hence, every experiment is uninformative in distinguishing the two hypotheses, and exponentially many measurements are required to achieve an algorithm output distribution under one hypothesis that is far in total variation from the other (Supplementary Theorems F.6 and F.8). The relationship between concentration of node-wise distributions and the final leaf output distribution is given by the martingale formalism developed in^16^. In model (ii), obtaining this bound requires quantifying the correlation between copies of the unknown state in terms of the size of the quantum memory and its lifetime, which we accomplish using a reformulation of the learning model in terms of Matrix Product States (Supplementary Lemma F.11).

For model (iii), nodes of the tree are joined by the state of the ancillary memory register after every measurement rather than a simple classical bitstring. Thus, we require a stronger approach: bounding the variation in entire root-to-leaf paths in the learning tree, following the approach from^10^. Using a probabilistic argument, we control the total variation of the leaf output distribution by bounding the number of paths which diverge substantially from the output distribution induced by the maximally mixed input state. To account for depolarizing noise in every copy of the state, we simplify the tensor-network analysis of^10^ via a technical argument which provides a cleaner form of the action of our noise channel. With this approach, we find that the contribution of the noise to the sample complexity decouples from the number of memory qubits; hence, even given *n* ancillary memory qubits, Ω((1−*λ*)^−*n*/3^) samples are still required in the noisy regime. We provide a two-copy algorithm which leverages noisy Bell sampling to match this asymptotic lower bound up to constants in the exponent, and give a single-copy classical shadow algorithm for the broader Pauli shadow tomography which accounts for noise by using shallow-circuit unitary ensembles. Up to polynomial factors and constants in the exponents, our upper and lower bounds for the Pauli-identification task exhibit the same exponential dependence on *n*, *k*, and the noise rate *λ*, matching the known noiseless bounds from^10,16^ as *λ* → 0 and diverging to infinity as *λ* → 1, where the task becomes information-theoretically impossible.

From the standpoint of near-term experiments, the most relevant regime is often not the asymptotic limit *n* → *∞* at fixed noise. Instead, the instance size *n* is effectively fixed by hardware, and the central question is how performance changes as other parameters, especially the noise rate *λ*, are improved. Our proof of Corollary 2.7 supports this viewpoint: for any fixed *n*, it gives an explicit *λ*-dependent separation between the optimal single-copy sample complexity *N*_*SC*_ and the two-copy sample complexity *N*_*TC*_, and thus provides a quantitative condition on *λ* for obtaining a meaningful two-copy advantage. This perspective is consistent with the experimental results of Ref. ^14^, which indicate that at modest instance sizes and sufficiently low noise, two-copy Pauli shadow protocols can substantially outperform single-copy baselines.

**Fig. 5:**
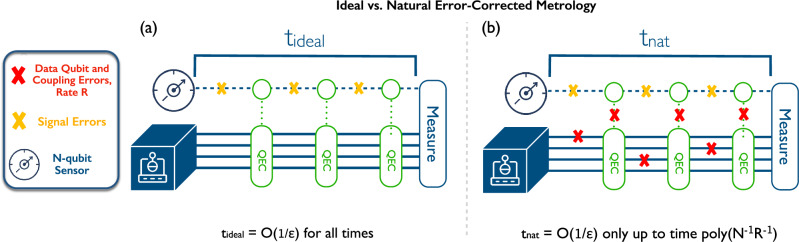
Schematic of error-corrected metrology in idealized and natural settings. **a** *Ideal error-corrected metrology*: interleaved quantum error correction (QEC) cycles protect an *N*-qubit probe during interrogation, sustaining Heisenberg-limited scaling in a noiseless sensing-control interface. **b** *Natural error-corrected metrology*: constant-rate faults at the sensing-control interface (red crosses, rate *R*) accumulate and restrict Heisenberg-limited performance to a finite sensing window (scaling to leading order as poly(*N*^−1^*R*^−1^)), beyond which the protocol crosses over to the Standard Quantum Limit.

Our noise- and resource-aware analyses extend beyond state learning. Figure 5 contrasts ideal error-corrected metrology, which can sustain Heisenberg-limited scaling indefinitely, with the NBQP setting, where noisy probe-control couplings confine Heisenberg-limited performance to a finite sensing window before a crossover to the Standard Quantum Limit. In Theorem 2.8, we bound this precision-time tradeoff of Heisenberg-limited (HL) single-parameter metrology protocols under an effective noise model similar to NBQP.

#### Theorem 2.8

(A threshold for Heisenberg-limited metrology, informal) Consider a single-parameter quantum sensing protocol in which a Hamiltonian *H*(*ω*) acts on an *N*-qubit probe, achieving Heisenberg-limited sensitivity when the sensing-control interface is noiseless. Under depolarizing noise of rate *R*(*τ*, *η*) (determined by the speed of control *τ* and native error strength *η*) at this interface, and without additional error mitigation, the protocol achieves the Heisenberg limit only up to a total evolution time inverse-polynomial in *NR*, beyond which it crosses over to the Standard Quantum Limit.

This result illustrates the value of noise-aware analysis beyond state preparation and measurement, namely that SPAM-robust metrology protocols can still fail under interstitial noise. It also highlights the connection between noisy quantum learning theory and practical metrology, demonstrating that even with access to quantum error correction, Heisenberg-limited metrological gains are delicate and tightly coupled to hardware constraints. The formal statement (Supplementary Theorem G.2) expresses the threshold sensing time in terms of control speed, native noise strength, and quantum memory size. When all parameters except *N* are Θ(1), HL sensitivity is essentially completely lost, as coherence-destroying errors accumulate on the same timescale as quantum control is executed in a single round. More generally, our bounds clarify the tradeoff between experimental resources and quantify how error rates must scale with *n* to sustain an HL protocol over a meaningful total time. For instance, an effective noise strength of *η* ~ 1/*N* and *Nητ* ≪ 1 enables HL sensitivity for *O*(1) time.

## Discussion

We have studied quantum-enhanced experiments in the presence of noise through the lens of quantum learning theory. Our results show that many of the most striking idealized quantum advantages in learning, property testing, and metrology disappear, or become inapplicable, once interstitial noise and unprotected oracle systems are taken into account. At the same time, we identified settings where advantages survive, often in a weakened but still meaningful form, and clarified how these surviving advantages depend on noise strength, memory resources, and problem structure.

Our work suggests several concrete directions for further development of noisy quantum learning theory. First, progress will require engaging more deeply with the physics of quantum many-body systems, rather than treating oracles as abstract. Many natural systems possess built-in robustness: renormalization-group structure and locality can protect long-range correlations^31,32,47–49^, and thermalizing dynamics can generate noise-resilient macroscopic observables^27–29^. It will be important to identify and characterize classes of states, channels, and observables whose relevant features are intrinsically stable under realistic error models, and to turn such structure into quantitatively sharp, physically natural examples of noisy quantum advantage.

Second, most known exponential quantum advantages in learning rest on highly entangled, global measurements, such as large-scale SWAP tests or Bell-basis measurements^10,11,46^, that are simultaneously fragile to noise and misaligned with the local-control constraints of real devices, which can couple to and manipulate experimental systems. This points toward a theory of noisy quantum learning under restricted resources, in which allowed operations may be shallow, geometrically local, few-qubit, or constrained to a small number of probe systems. Quantum probe tomography^50^ is an example of this philosophy, wherein a small number of probes interacting locally with a large system can still extract global information under noise. It will be important to understand systematically which restricted-access models admit robust (even if only polynomial) advantages over conventional experiments, and how those advantages trade off against architectural constraints.

There is a close connection between noisy quantum learning and quantum sensing. Many sensing protocols can be viewed as learning problems about Hamiltonian parameters or state observables under stringent access and noise constraints^13,51,52^. Some ambitious proposals, such as quantum computation-enhanced sensing based on deep coherent control^13,52^ or Grover-type oracles^53^, lose their asymptotic advantage in the NBQP noise model. This raises a quantitative question rather than a purely asymptotic one: how does the achievable quantum advantage degrade as a function of the noise rate, circuit depth, and available memory? Corollary 2.7, together with related work such as Ref. ^46^, suggests that in realistic noise regimes one should expect noise-dependent polynomial improvements rather than noise-independent exponential ones, but those polynomial advantages can still be practically meaningful.

Taken together, these directions aim at a more operational understanding of what fault-tolerant quantum computers can teach us about real-world quantum systems that are neither fully protected nor perfectly characterized. Our results indicate that quantum advantages do remain available in this regime, but they are more delicate, more problem-dependent, and more tightly coupled to physical structure than in idealized oracle models. Developing a mature noisy quantum learning theory along these lines should inform both the design of near- and medium-term experiments and the long-term role of quantum computers as scientific instruments.

## Supplementary information

Supplementary Information

Transparent Peer Review file

## Data Availability

No data was generated in this work.

## References

[1] Aaronson, S. The learnability of quantum states. *Proc. R. Soc. A: Math., Phys. Eng. Sci.* **463**, 3089–3114 (2007).

[2] Haah, J., Harrow, A. W., Ji, Z., Wu, X. & Yu, N. Sample-optimal tomography of quantum states. *IEEE Trans. Inf. Theory* **63**, 5628–5641 (2017).

[3] Wang, J. et al. Experimental quantum Hamiltonian learning. *Nat. Phys.* **13**, 551–555 (2017).

[4] Aaronson, S. Shadow Tomography of Quantum States. In *Proceedings of the 50th Annual ACM SIGACT Symposium on Theory of Computing (STOC 2018).* (2018).

[5] Arunachalam, S. & de Wolf, R. Optimal Quantum Sample Complexity of Learning Algorithms. *J. Mach. Learn. Res.* **19**, 1–36 (2018).

[6] Aaronson, S., Chen, X., Hazan, E., Kale, S. & Nayak, A. Online learning of quantum states. In *Advances in Neural Information Processing Systems.* **31**, (2018).

[7] Huang, H.-Y., Kueng, R. & Preskill, J. Predicting many properties of a quantum system from very few measurements. *Nat. Phys.* **16**, 1050–1057 (2020).

[8] Cotler, J. & Wilczek, F. Quantum overlapping tomography. *Phys. Rev. Lett.* **124**, 100401 (2020). 32216420 10.1103/PhysRevLett.124.100401

[9] Huang, H.-Y., Kueng, R. & Preskill, J. Information-theoretic bounds on quantum advantage in machine learning. *Phys. Rev. Lett.*10.1103/PhysRevLett.126.190505 (2021). 10.1103/PhysRevLett.126.190505 34047595

[10] Chen, S., Cotler, J., Huang, H.-Y. & Li, J. Exponential separations between learning with and without quantum memory. In *2021 IEEE 62nd Annual Symposium on Foundations of Computer Science (FOCS)*. 574–585 (IEEE, 2022).

[11] Aharonov, D., Cotler, J. & Qi, X.-L. Quantum algorithmic measurement. *Nat. Commun.*10.1038/s41467-021-27922-0 (2022). 10.1038/s41467-021-27922-0 PMC8850572 35173160

[12] Chen, S., Cotler, J., Huang, H.-Y. & Li, J. The complexity of NISQ. *Nat. Commun.* **14**, 6001 (2023). 37752125 10.1038/s41467-023-41217-6 PMC10522708

[13] Huang, H.-Y., Tong, Y., Fang, D. & Su, Y. Learning Many-Body Hamiltonians with Heisenberg-Limited Scaling. *Phys. Rev. Lett.*10.1103/PhysRevLett.130.200403 (2023). 10.1103/PhysRevLett.130.200403 37267566

[14] Huang, H.-Y. et al. Quantum advantage in learning from experiments. *Science* **376**, 1182–1186 (2022). 35679419 10.1126/science.abn7293

[15] Nöller, J., Tran, V. T., Gachechiladze, M. & Kueng, R. An infinite hierarchy of multi-copy quantum learning tasks. *arXiv* https://arxiv.org/abs/2510.08070 (2025).

[16] Chen, S., Gong, W. & Ye, Q. Optimal tradeoffs for estimating Pauli observables. In *2024 IEEE 65th Annual Symposium on Foundations of Computer Science (FOCS)*. 1086–1105 (IEEE, 2024).

[17] King, R., Gosset, D., Kothari, R. & Babbush, R. Triply Efficient Shadow Tomography. *PRX Quantum*10.1103/PRXQuantum.6.010336 (2025).

[18] Schollwöck, U. The density-matrix renormalization group in the age of matrix product states. *Ann. Phys.* **326**, 96–192 (2011).

[19] Begušić, T., Gray, J. & Chan, G. K.-L. Fast and converged classical simulations of evidence for the utility of quantum computing before fault tolerance. *Sci. Adv.* **10**, eadk4321 (2024). 10.1126/sciadv.adk4321 PMC12697566 38232163

[20] Gong, W., Haferkamp, J., Ye, Q. & Zhang, Z. On the sample complexity of purity and inner product estimation. *arXiv* https://arxiv.org/abs/2410.12712. (2024).

[21] Harrow, A. W. & Montanaro, A. Testing product states, quantum Merlin-Arthur games and tensor optimization. *J. ACM.* **60**, 1–43 (2013).

[22] Lloyd, S., Mohseni, M. & Rebentrost, P. Quantum principal component analysis. *Nat. Phys.* **10**, 631–633 (2014).

[23] Bădescu, C., O’Donnell, R. & Wright, J. Quantum state certification. In *Proceedings of the 51st Annual ACM SIGACT Symposium on Theory of Computing*. 503–514 (2019).

[24] Montanaro, A. & de Wolf, R. A survey of quantum property testing. *Theory Comput. Libr. Grad. Surv*. **7**, 1–81 (2016).

[25] Aharonov, D., Ben-Or, M., Impagliazzo, R. & Nisan, N. Limitations of noisy reversible computation. *arXiv* https://arxiv.org/abs/quant-ph/9611028 (1996).

[26] Kannan, I., Putterman, H. & Cotler, J. Exponential speedups in fault-tolerant processing of quantum experiments. *arXiv*10.48550/arXiv.2605.02057 (2026).

[27] Chesi, S., Röthlisberger, B. & Loss, D. Self-correcting quantum memory in a thermal environment. *Phys. Rev. A*10.1103/PhysRevA.82.022305 (2010).

[28] Brandão, F. G., Crosson, E., Sahinoglu, M. B. & Bowen, J. Quantum error correcting codes in eigenstates of translation-invariant spin chains. *Phys. Rev. Lett.*10.1103/PhysRevLett.123.110502 (2019). 10.1103/PhysRevLett.123.110502 31573226

[29] Kastoryano, M. J., Kristensen, L. B., Chen, C.-F. & Gilyén, A. A little bit of self-correction. *Quantum* **9**, 1820 (2025).

[30] Pastawski, F., Yoshida, B., Harlow, D. & Preskill, J. Holographic quantum error-correcting codes: toy models for the bulk/boundary correspondence. *J. High Energy Phys.*10.1007/JHEP06(2015)149 (2015).

[31] Kim, I. H. & Kastoryano, M. J. Entanglement renormalization, quantum error correction, and bulk causality. *J. High Energy Phys.*10.1007/JHEP04(2017)040 (2017).

[32] Lake, E., Balasubramanian, S. & Choi, S. Exact Quantum Algorithms for Quantum Phase Recognition: Renormalization Group and Error Correction. *PRX Quantum*10.1103/PRXQuantum.6.010350 (2025).

[33] Chia, N.-H., Chung, K.-M. & Lai, C.-Y. On the need for large quantum depth. *J. ACM* **70**, 1–38 (2023).

[34] Aharonov, D. & Ben-Or, M. Fault-tolerant quantum computation with constant error. In *Proceedings of the twenty-ninth annual ACM symposium on Theory of Computing*. 176–188 (1997).

[35] Simon, D. R. On the power of quantum computation. In *Proceedings 35th Annual Symposium on Foundations of Computer Science*. 116–123 (1994).

[36] Fenner, S., Green, F., Homer, S. & Zhang, Y. Bounds on the power of constant-depth quantum circuits. In *International Symposium on Fundamentals of Computation Theory*. https://link.springer.com/chapter/10.1007/11537311_5 (Springer, 2005).

[37] Hoyer, P. & Spalek, R. Quantum fan-out is powerful. *Theory Comput.* **1**, 81–103 (2005).

[38] Haferkamp, J., Faist, P., Kothakonda, N. B. T., Eisert, J. & Yunger Halpern, N. Linear growth of quantum circuit complexity. *Nat. Phys.* **18**, 528–532 (2022).

[39] Arora, A. S. et al. Quantum depth in the random oracle model. In *Proceedings of the 55th Annual ACM Symposium on Theory of Computing*. 1111–1124 (2023).

[40] Bubeck, S., Chen, S. & Li, J. Entanglement is necessary for optimal quantum property testing. In *2020 IEEE 61st Annual Symposium on Foundations of Computer Science (FOCS)*. 692–703 (IEEE, 2020).

[41] Cincio, L., Subasi, Y., Sornborger, A. T. & Coles, P. J. Learning the quantum algorithm for state overlap. *N. J. Phys.* **20**, 113022 (2018).

[42] Maldacena, J. The large-n limit of superconformal field theories and supergravity. *Int. J. Theor. Phys.* **38**, 1113–1133 (1999).

[43] Jahn, A. & Eisert, J. Holographic tensor network models and quantum error correction: a topical review. *Quantum Sci. Technol.* **6**, 033002 (2021).

[44] Harris, R. J., Coupe, E., McMahon, N. A., Brennen, G. K. & Stace, T. M. Decoding holographic codes with an integer optimization decoder. *Phys. Rev. A*10.1103/PhysRevA.102.062417 (2020).

[45] Farrelly, T., Harris, R. J., McMahon, N. A. & Stace, T. M. Tensor-Network Codes. *Phys. Rev. Lett.*10.1103/PhysRevLett.127.040507 (2021). 10.1103/PhysRevLett.127.040507 34355960

[46] Huang, H.-Y., Flammia, S. T. & Preskill, J. Foundations for learning from noisy quantum experiments. *arXiv* https://arxiv.org/abs/2204.13691 (2022).

[47] Furuya, K., Lashkari, N. & Ouseph, S. Real-space RG, error correction and Petz map. *J. High Energy Phys.*10.1007/JHEP01(2022)170 (2022).

[48] Furuya, K., Lashkari, N. & Moosa, M. Renormalization group and approximate error correction. *Phys. Rev. D*10.1103/PhysRevD.106.105007 (2022).

[49] Goldman, S., Lashkari, N. & Leigh, R. G. A Lindbladian for exact renormalization of density operators in QFT. *arXiv* https://arxiv.org/abs/2410.16582 (2024).

[50] Chen, S., Cotler, J. & Huang, H.-Y. Quantum probe tomography. *arXiv* https://arxiv.org/abs/2510.08499. (2025).

[51] Zhou, S., Zhang, M., Preskill, J. & Jiang, L. Achieving the Heisenberg limit in quantum metrology using quantum error correction. *Nat. Commun.*10.1038/s41467-017-02510-3 (2018). 10.1038/s41467-017-02510-3 PMC5758555 29311599

[52] Hu, H.-Y. et al. Ansatz-free Hamiltonian learning with Heisenberg-limited scaling. *PRX Quantum*10.1103/j7b8-pb77 (2025).

[53] Allen, R. R., Machado, F., Chuang, I. L., Huang, H.-Y. & Choi, S. Quantum computing enhanced sensing. *arXiv* https://arxiv.org/abs/2501.07625 (2025).

