# Supplementary material for: Noisy quantum learning theory
Source: Nat Commun. 2026 May 29;17:6979. doi: 10.1038/s41467-026-73693-x (PMC13392386; doi:10.1038/s41467-026-73693-x)
Supplement: Supplementary file 2 — Transparent Peer Review file [file 41467_2026_73693_MOESM2_ESM.pdf]

Peer Review File

Noisy Quantum Learning Theory

Corresponding Author: Mr Ishaan Kannan

This file contains all reviewer reports in order by version, followed by all author rebuttals in order by version.

Version 0:

Reviewer comments:

Reviewer #1

(Remarks to the Author)
This manuscript studies quantum learning in a noisy setting where a fault-tolerant quantum computer interacts with an uncharacterized external quantum system through noisy couplings. The authors introduce the complexity class NBQP to formalize this regime, establish oracle separations between BQP, NBQP, and NISQ, and then apply the framework to several representative tasks, including purity testing, shadow tomography, and quantum metrology. The paper also presents a positive example, based on a HaPPY-code-inspired setting, showing that meaningful quantum advantages can survive when the target system itself has suitable noise-robust structures.

I find the paper timely and important. It partly solved a question I had for a while. Its main conceptual contribution is to clarify that many well-known quantum learning advantages rely on fragile coherent primitives, and that these advantages can collapse once one models the physically realistic asymmetry between a protected quantum processor and an unprotected target system. At the same time, the paper does not deliver a purely negative message: it also identifies a route by which robust advantages may persist, namely through structured physical systems with intrinsic error-resilient features. This balance between no-go style results and constructive insight.

The technical scope is also impressive. The oracle separations give a clean complexity-theoretic foundation for the distinction between idealized and physically realistic quantum learning. The purity-testing result is particularly striking, as it shows that the celebrated two-copy advantage can disappear under local noise. The shadow tomography results further strengthen the paper by providing a more quantitative tradeoff picture rather than a purely binary conclusion. Overall, I believe the paper will be of broad interest to researchers in quantum information, quantum learning, quantum sensing, and fault-tolerant quantum computing.

I support publication in Nature Communications, subject to minor revision. My main suggestions concern presentation and scope clarification:

1. The scope of the negative results could be clarified more explicitly in the Introduction and possibly revisited in the Outlook. As written, the paper strongly emphasizes the fragility of ideal quantum learning advantages under noisy couplings, but it would help to state even more plainly that the results apply to a broad but still specific noisy-query framework, rather than ruling out all hybrid fault-tolerant/non-fault-tolerant architectures.

2. The assumptions behind the NBQP model should be summarized more transparently in the main text, especially around Section 2.1. It would be useful to separate more clearly which statements rely specifically on local depolarizing noise and which extend to more general local stochastic noise models.

3. The paper would benefit from a short roadmap paragraph near the end of the Introduction or at the beginning of Section 2, explaining which technical ingredients underlie which main results, for example, oracle constructions for the complexity separations, learning-tree methods for lower bounds, and noisy Bell/swap-type constructions for the upper bounds.

4. The discussion of the shadow-tomography tradeoff in Section 2.3 could be strengthened by adding a brief schematic or numerical illustration. Since this part of the paper gives one of the most operationally interesting noise-dependent tradeoff pictures, even a simple representative scaling plot would make the message more accessible.

In summary, I find this to be a strong paper with substantial conceptual and technical value. With some revision to improve clarity and positioning, I believe it is well suited for publication in Nature Communications.

Reviewer #2

(Remarks to the Author)
This paper introduced noise BQP complexity class (NBQP) to model fault-tolerant quantum computers that still face noise when interacting with noisy environmental systems (represented by noisy oracles in the model). Furthermore, it shows oracle separations between NBQP and other related classes (that BQP and NISQ); In particular, it uses lifted SImon's problem and shuffling Simon's problem from previous results to demonstrate such separations.

This work also explores the advantages of a noisy quantum model used for NBQP, though the findings do not directly relate to or derive from NBQP class. (This is due to the fact that NBQP is for languages.) This model incorporates a depolarizing channel noise after each quantum circuit layer. It permits mid-circuit measurements, the introduction of fresh qubits, and all standard quantum algorithm operations. Since this model is capable of fault-tolerant quantum computing, its distinction primarily lies in its interaction with the oracle or noisy data.

The study extensively investigates how noisy data impacts efficiency across various applications within this model. Specifically, it details: a) The absence of quantum advantage for purity testing using two-copy measurement. b) The sample complexity for noisy Pauli shadow tomography. c) The conditions under which this model can achieve the Heisenberg limit for single-parameter quantum sensing.

Overall, this paper provides a comprehensive study on the potential applications of the proposed noisy quantum computation, formalizes relevant complexity classes, and identifies the inherent limitations of such models. On the positive side, the authors introduce a plausible framework for utilizing future quantum computers to process noisy data, yielding several compelling results. However, the manuscript appears to rely heavily on techniques and concepts from existing literature. It lacks sufficient original insights or systematic conceptual advancements that significantly build upon prior work. Consequently, I think the level of conceptual novelty and depth to be below the threshold for publication in Nature Communications.

Open Access This Peer Review File is licensed under a Creative Commons Attribution 4.0 International License, which permits use, sharing, adaptation, distribution and reproduction in any medium or format, as long as you give appropriate credit to the original author(s) and the source, provide a link to the Creative Commons license, and indicate if changes were made.
In cases where reviewers are anonymous, credit should be given to 'Anonymous Referee' and the source.
The images or other third party material in this Peer Review File are included in the article's Creative Commons license, unless indicated otherwise in a credit line to the material. If material is not included in the article's Creative Commons license and your intended use is not permitted by statutory regulation or exceeds the permitted use, you will need to obtain permission directly from the copyright holder.
To view a copy of this license, visit https://creativecommons.org/licenses/by/4.0/

Reviewer #1 (Remarks to the Author):

This manuscript studies quantum learning in a noisy setting where a fault-tolerant quantum computer interacts with an uncharacterized external quantum system through noisy couplings. The authors introduce the complexity class NBQP to formalize this regime, establish oracle separations between BQP, NBQP, and NISQ, and then apply the framework to several representative tasks, including purity testing, shadow tomography, and quantum metrology. The paper also presents a positive example, based on a HaPPY-code-inspired setting, showing that meaningful quantum advantages can survive when the target system itself has suitable noise-robust structures.

I find the paper timely and important. It partly solved a question I had for a while. Its main conceptual contribution is to clarify that many well-known quantum learning advantages rely on fragile coherent primitives, and that these advantages can collapse once one models the physically realistic asymmetry between a protected quantum processor and an unprotected target system. At the same time, the paper does not deliver a purely negative message: it also identifies a route by which robust advantages may persist, namely through structured physical systems with intrinsic error-resilient features. This balance between no-go style results and constructive insight.

Author response: *We thank the reviewer for their positive remarks on the balance between no-go results and nuanced directions towards practical, noise-aware quantum learning, and we are glad that the conceptual takeaways we aimed to convey were well-received.*

The technical scope is also impressive. The oracle separations give a clean complexity-theoretic foundation for the distinction between idealized and physically realistic quantum learning. The purity-testing result is particularly striking, as it shows that the celebrated two-copy advantage can disappear under local noise. The shadow tomography results further strengthen the paper by providing a more quantitative tradeoff picture rather than a purely binary conclusion. Overall, I believe the paper will be of broad interest to researchers in quantum information, quantum learning, quantum sensing, and fault-tolerant quantum computing.

Author response: *We thank the reviewer for their positive comments on the technical scope of our work, and in particular on the noise-aware lower bound methods we introduce. We are encouraged that the reviewer finds our manuscript to be of broad interest.*

I support publication in Nature Communications, subject to minor revision. My main suggestions concern presentation and scope clarification:

1. The scope of the negative results could be clarified more explicitly in the Introduction and possibly revisited in the Outlook. As written, the paper strongly emphasizes the fragility of ideal quantum learning advantages under noisy couplings, but it would help to state even more plainly that the results apply to a broad but still specific noisy-query framework, rather than ruling out all hybrid fault-tolerant/non-fault-tolerant architectures.

Author response: *We thank the reviewer for their comments on our noisy learning model. We would like to note a nuance related to the interpretation of our computational model of λ-noisy circuits and its relation to real-world learning protocols. Indeed, particular fault-tolerant architectures and hardwares may have complex noise models and methods of coupling to physical systems. However, while our models utilize a constant local depolarizing noise, our lower bounds automatically apply to all such complex architectures assuming that each qubit is subject to some constant, nonzero amount of noise along Pauli X, Y, and Z directions. Correlated or adversarial noise models are also encompassed, as they are stronger noise models. Hence, despite the abstraction to a computational model, our results apply to a wide array of hybrid architectures. This does not, however, include weaker noise models where hardwares are deliberately biased to suppress particular noise quadratures. To emphasize that noise in all Bloch-sphere directions is the primary assumption of our framework, we add the following sentence to our Introduction section:*

"These results pertain to a broad framework which we introduce to model noisy quantum experiments, in which the learner is subjected to repeated depolarizing noise upon querying natural oracle systems but is permitted to use any (possibly adaptive) strategy that can include quantum error-correction."

We have also lightly restructured the Introduction and Results sections to add specificity and clarify the scope of negative results.

2. The assumptions behind the NBQP model should be summarized more transparently in the main text, especially around Section 2.1. It would be useful to separate more clearly which statements rely specifically on local depolarizing noise and which extend to more general local stochastic noise models.

Author response: *We thank the reviewer for their comments, and agree that further details behind the NBQP model would provide clarity to the main text. We have added a paragraph after Definition 2.1 which provides a more formal description of the computational model in words, and comparison to the NISQ model in previous work.*

3. The paper would benefit from a short roadmap paragraph near the end of the Introduction or at the beginning of Section 2, explaining which technical ingredients underlie which main results, for example, oracle constructions for the complexity separations, learning-tree methods for lower bounds, and noisy Bell/swap-type constructions for the upper bounds.

Author response: *We thank the reviewer for the suggestion to improve the readability of our manuscript. We have added a thorough roadmap of the main results and the technical ingredients used, as suggested.*

4. The discussion of the shadow-tomography tradeoff in Section 2.3 could be strengthened by adding a brief schematic or numerical illustration. Since this part of the paper gives one of the most operationally interesting noise-dependent tradeoff pictures, even a simple representative scaling plot would make the message more accessible.

Author response: *We thank the reviewer for the suggestion and agree that this may strengthen one of the most interesting tradeoff pictures of our work. We have added Figure 4, which schematically plots the*

different regimes of advantage. We have also restructured Corollary 2.7 and its discussion in the supplementary material to emphasize the regimes of exponential vs. polynomial advantage, and how these depend jointly on noise and instance size.

In summary, I find this to be a strong paper with substantial conceptual and technical value. With some revision to improve clarity and positioning, I believe it is well suited for publication in Nature Communications.

Author response: *We thank the reviewer for their detailed comments on our work, which has helped us strengthen its presentation.*

—---
Reviewer #2 (Remarks to the Author):

This paper introduced noise BQP complexity class (NBQP) to model fault-tolerant quantum computers that still face noise when interacting with noisy environmental systems (represented by noisy oracles in the model). Furthermore, it shows oracle separations between NBQP and other related classes (that BQP and NISQ); In particular, it uses lifted Simon's problem and shuffling Simon's problem from previous results to demonstrate such separations.

This work also explores the advantages of a noisy quantum model used for NBQP, though the findings do not directly relate to or derive from NBQP class. (This is due to the fact that NBQP is for languages.) This model incorporates a depolarizing channel noise after each quantum circuit layer. It permits mid-circuit measurements, the introduction of fresh qubits, and all standard quantum algorithm operations. Since this model is capable of fault-tolerant quantum computing, its distinction primarily lies in its interaction with the oracle or noisy data.

Author response: *We thank the reviewer for their detailed remarks on the contents of our work. While we agree that the quantum learning separations for SWAP tests and Bell sampling do not formally fit into complexity-theoretic language, we note that the circuits used in the NBQP model are contained within the class of protocols against which our work proves lower bounds. While the complexity class itself is used to establish broader and more abstract statements about the power of realistic noisy quantum experiments, the same mathematical tools and computational models are used throughout all results in our work.*

The study extensively investigates how noisy data impacts efficiency across various applications within this model. Specifically, it details: a) The absence of quantum advantage for purity testing using two-copy measurement. b) The sample complexity for noisy Pauli shadow tomography. c) The conditions under which this model can achieve the Heisenberg limit for single-parameter quantum sensing.

Overall, this paper provides a comprehensive study on the potential applications of the proposed noisy quantum computation, formalizes relevant complexity classes, and identifies the inherent limitations of such models. On the positive side, the authors introduce a plausible framework for utilizing future quantum computers to process noisy data, yielding several compelling results. However, the manuscript

appears to rely heavily on techniques and concepts from existing literature. It lacks sufficient original insights or systematic conceptual advancements that significantly build upon prior work. Consequently, I think the level of conceptual novelty and depth to be below the threshold for publication in Nature Communications.

Author response: *We thank the reviewer for their comments, and are encouraged that our results are received as both comprehensive and compelling. Our work draws upon and combines several contemporary developments in quantum learning theory, particularly related to information-theoretic lower bounds for adaptive learning strategies. While the "learning tree" formalism is well established in existing literature, we believe that our work advances this lower-bound machinery towards applicability in more realistic settings.*

Previously, learning trees have been limited in their ability to produce lower bounds that simultaneously account for many quantum resources, and incorporating bounded quantum memory was previously cumbersome. As a result, the obtained bounds were purely exponential in instance size, and do not capture further nuanced features of the underlying problem. Our work improves on these limitations in multiple ways that we believe may be more broadly useful in quantum learning and sensing.

1. *Nearly all previous learning tree results exhibit a separation governed only by the number of copies measured simultaneously. However, to our knowledge, no prior works have established nontrivial lower bounds even when the requisite number of copies (in the idealized setting) is provided. As such, it has been challenging to rigorously understand the power of other resources, or the challenge posed by experimental constraints. Our work addresses this gap. To prove our purity-testing bound, we introduce a new learning tree methodology that goes beyond the usual "node-based" and "path-based" frameworks established in prior work — in fact, one quickly finds that the standard form of these approaches fails for the desired bound. Our method initially leverages the learning tree formalism to restructure the task, then achieves the desired result by proving a bound on the operator norm on a particular operator that encodes the distinguishability between two hypotheses. To our knowledge, this "operator-based" approach is not present in prior work, and it generalizes to various one-vs-many and many-vs-many hypothesis testing problems. This is a transferable approach that can be used to great effect in the setting where the limiting quantum resource is not the number of replicas, but another practically-motivated restriction on the learning protocol such as noise.*
2. *Most previous works exhibit sharp exponential lower bounds, but this does not immediately provide insight on the structure of the problem in the constrained setting. Our purity-testing separation is surprising in this regard, as we show that even one layer of noise exponentially degrades the idealized O(1) sample complexity. Our bound is closely tied to the algebraic structure of the purity testing problem, and one can show that the strange* $(4/(1+3(1-\lambda))^n$ *bound we prove comes from the operator norm of the 2-cycle (SWAP) operator. In this work, we do not discuss an upper bound as the primary focus is degradation of the SWAP primitive, but it is possible to show that this parametric structure is actually optimal for the original hypothesis testing problem in the presence of noise. Similarly, our Pauli tomography lower bound simultaneously considers tradeoffs between instance size, quantum coherence, and memory, and has a form that parametrically matches the upper bound. As such, our approach suggests a strategy to obtain insights on obtaining optimal learning protocols in constrained, noisy settings.*

3. *Lower bounds that incorporate sustained quantum memory previously utilized complex tensor-network computations that make the source of the bound difficult to interpret. In our bounded-memory Pauli separation, we simplify previous tensor-network machinery used by Chen, Cotler, Huang Li '21 by identifying that the operator which controls the total-variation divergence has particular structure that decomposes into symmetric and antisymmetric components. This strategy is not unique to our result; in fact, it is possible to simplify other bounds in the quantum learning literature using simple extensions of this observation.*
